# Surviving spinal cord injury in low income countries

**DOI:** 10.4102/ajod.v3i2.80

**Published:** 2014-08-26

**Authors:** Tone Øderud

**Affiliations:** 1 SINTEF Technology and Society, Oslo, Norway

## Abstract

**Background:**

Mortality rates from injuries are higher for people from poorer economic backgrounds than those with higher incomes (according to the World Health Organization [WHO]), and health care professionals and organisations dealing with people with disabilities experience that individuals with spinal cord injury (SCI) in low income countries face serious challenges in their daily lives.

**Objectives:**

The aims of this study were to explore life expectancy (life expectancy is the average remaining years of life of an individual) and the situation of persons living with SCI in low income settings.

**Method:**

Literature studies and qualitative methods were used. Qualitative data was collected through semi-structured interviews with 23 informants from four study sites in Zimbabwe representing persons with SCI, their relatives and rehabilitation professionals.

**Results:**

There are few publications available about life expectancy and the daily life of persons with SCI in low income countries. Those few publications identified and the study findings confirm that individuals with SCI are experiencing a high occurrence of pressure sores and urinary tract infections leading to unnecessary suffering, often causing premature death. Pain and depression are frequently reported and stigma and negative attitudes are experienced in society. Lack of appropriate wheelchairs and services, limited knowledge about SCI amongst health care staff, limited access to health care and rehabilitation services, loss of employment and lack of financial resources worsen the daily challenges.

**Conclusion:**

The study indicates that life expectancy for individuals with SCI in low income settings is shorter than for the average population and also with respect to individuals with SCI in high income countries. Poverty worsened the situation for individuals with SCI, creating barriers that increase the risk of contracting harmful pressure sores and infections leading to premature death. Further explorations on mortality and how individuals with SCI and their families in low income settings are coping in their daily life are required to provide comprehensive evidences.

## Introduction

Globally 5.8 million people die each year from injuries (WHO 2010:2). Injuries kill more people than HIV and AIDS and malaria combined, and road traffic accidents account for about a quarter of deaths from injuries (WHO 2010:2). However, many people survive their injuries, and have to live with a permanent disability, often a spinal cord injury (SCI) (WHO 2010:6).

More than 90% of deaths that result from injury occur in low and middle income countries, and mortality rates from injuries are higher for people from poorer economic backgrounds than those with a higher income (WHO 2010:10). SCI varies in aetiology, and nations with similar economies tend to have similar features and incidence categories (Ackery, Tator & Krassioukov 2004:1355). Poorer people have an increased risk of injuries, and they are hardest hit by the financial pressure resulting from injuries (WHO 2010:10).

The scope of the study is to highlight the situation of persons living with SCI in low income countries, and to identify mechanisms facilitating survival and factors that might lead to death. The study addresses research topics that are derived from the gaps uncovered in the review of literature and from experiences in the field. Four study sites in Zimbabwe were selected representing urban and rural low income areas. The specific research objectives are to explore life expectancy and how individuals with SCI cope in their daily life. It explores the daily challenges experienced by persons with SCI and their families after returning home to their local communities. Life expectancy for persons with SCI from low income countries is expected to be lower than for persons with SCI from high income countries.

When interviewed on 19 September 2006, the executive director of the Disabled Women Support Organisation in Zimbabwe, Ms Gladys Charowa, illustrated the situation as follows:

‘We were 19 people being rehabilitated in 2001 and discharged in 2003. I am now the only person alive. The rest have died because of pressure sores. If someone can’t afford a wheelchair and is using a wheelbarrow and doesn’t have a cushion what do you expect?’

The living conditions studies amongst people with disabilities in the southern African region find that individuals with disabilities and their households are worse off on many important indicators of living conditions, and they often live without optimal technical, medical or social support that could improve their level of living condition considerably (Eide *et al*. 2011). It is estimated that 80% of the world’s population of people with disabilities live in low income countries (WHO 2007:1), and as a consequence of poverty, many people with disabilities are likely to live with limited access to appropriate health care and rehabilitation services (WHO 2006; World Bank 2001:152).

## Research Method and design

### Review of literature

A limited literature review using PubMed was carried out, including the option of search for ‘related article’ and searching the publications’ reference lists. The search was limited to articles and abstracts published in English from 1992 to 2010. Search terms used were, ‘spinal cord injury’ cross-indexed with ‘developing countries’, ‘low income countries’, ‘morbidity’, ’mortality’, ‘life expectancy’, ‘epidemiology’, ‘incidence’, ‘prevalence’ and ‘quality of life’. The identified articles were screened for relevance to this study. In addition the World Bank and WHO’s websites were used to obtain demographic, economic and health statistics, especially from low income countries.

The review of literature revealed numerous studies on life expectancy and epidemiology of SCI from high income countries (Burt 2004; DeVivo, Krause & Lammertse 1999; Dryden *et al*. 2003; Hagen *et al*. 2010; Krause *et al*. 2008; Lidal *et al*. 2007; O’Connor 2005; Soden *et al*. 2000; Strauss *et al*. 2006; Whiteneck *et al*. 1992; Yeo *et al*. 1998). However, there were few epidemiological studies of life expectancy and SCI in low income countries, except for studies from Zimbabwe (Levy *et al*. 1998), Bangladesh (Hoque, Grangeon & Reed 1999), Nigeria (Iwegbu 1983; Nwadinigwe, Iloabuchi & Nwabude 2004) and Sierra Leone (Gosselin & Coppotelli 2005). A qualitative long-term follow-up research study from Botswana (Ingstad & Whyte 2007) experienced that 12 out of 46 persons (26%) with disabilities had died within a life span of 16 years (1985/86 to 2002), and two men who became paraplegic after a mine accident, had died from infections.

### Qualitative research methods

A qualitative study highlighting the situation experienced by people with SCI and their families in Zimbabwe was conducted. Qualitative research methods are used in the exploration of meanings of social phenomena as experienced by individuals themselves in their natural context (Malterud 2001a). Qualitative research involves systematic collection, organisation and interpretation of textual material derived from talks or observations (Malterud 2001b). A heterogeneous group of 23 informants (I) were recruited from urban areas of Bulawayo and Harare and from rural areas of Tsholotsho and Binga in Zimbabwe. Data was collected through semi-structured interviews, and the informants were interviewed in their local environment. Purposive sampling was used to ensure maximum variability of experiences and different aspects of living with a SCI (Domholdt 2005). Representatives from disabled peoples’ organisations and rehabilitation professionals from local hospitals assisted the researcher to identify and interview the individual informants in the local communities. The study sample informants included persons with SCI (11), families having had one family member with SCI passing away (5), representatives from organisations of people with SCI (2) and rehabilitation professionals (5) from the governmental health care system (two rehabilitation technicians, one nurse and two occupational therapists). Three interview guides were designed, one for persons with SCI, one for relatives of persons with SCI who had passed away and one for rehabilitation professionals. All interview guides had open-ended questions describing the main topics and themes.

### Verbal autopsy

Verbal autopsy is a method for identifying the cause of death based on interviews with relatives or other caregivers, and is often the only way to establish the causes of death in areas where civil registration and death certification systems are weak (Setel *et al*. 2006:693). For this study we applied elements from verbal autopsy for identifying causes of death and factors facilitating the survival of persons with SCI. Standard validated verbal autopsy questionnaires were modified for the interview guide for semi-structured interviews of relatives and caregivers of persons with SCI who had passed away.

Data collected from interviews, observations during interviews and field notes were transcribed into written text. The data was coded into given categories in a hierarchic or tree structure. Being an iterative process data analysis started together with the data collection. When analysing data the important cultural and contextual differences and the perception of disability (Ingstad & Whyte 2007) that might be defined and understood differently in different settings were kept in mind.

### Study limitations

The findings presented are limited to the 23 informants from four study settings in one low income country: the literature however confirms similar findings from other low income countries (Gosselin & Coppotelli 2005; Hoque *et al*. 1999; Iwegbu 1983; Levy *et al*. 1998; Nwadinigwe *et al*. 2004). The findings could serve as a basis for a larger research study covering more countries, and it would add value if comparative studies could be undertaken in similar African countries. This was not possible within the scope of this study. Data was coded and analysed by the author, and greater rigour might have been achieved if the data was coded and analysed by more than one researcher.

## Ethical consideration

Permission to conduct the study was granted by the ministry of health and child welfare, Zimbabwe. Participation in the study was voluntary, and the informants were at liberty to withdraw from the interview at any time. All informants were informed about the objectives of the study, and informed consent was signed prior to the interviews. Participating in the study did not adversely influence the informants’ access to health care or wheelchair services. Data gathered was treated confidentially. All names used in this article are fictitious in order to protect the informants’^1^ identity.

## Results

### Aetiology of spinal cord injury

The recent global estimate of traumatic SCI incidence^2^ ranges between 10.4 and 83 cases per million inhabitants per year (Wyndaele & Wyndaele 2006:525), and the prevalence^3^ of SCI ranges between 223 and 755 cases per million inhabitants (Wyndaele & Wyndaele 2006:525).

SCI is caused by damage to the spinal cord that carries sensation and motor signals to and from the brain, and affects sensation and voluntary movement below the level of the injury (Lin *et al*. 2003). The loss of function depends on the severity of injury and the impact on the spinal cord. Functions that might be affected are bladder and bowel control, pain, spasm, sexual function, blood pressure, heart rate, digestion, temperature control, sweating and other autonomic functions (Lin *et al*. 2003). Traumatic SCI might result from road traffic accidents, falls, violence, sporting accidents, war injuries and work related accidents. Non-traumatic SCI can occur from tumours, spinal tuberculosis, ischaemia, development disorders like spina bifida and other neuro-degenerative diseases (Lin *et al*. 2003).

SCI varies in aetiology, male-to-female ratio, age distribution and complications with reference to country and region, depending on economic, social and cultural factors (Ackery *et al*. 2004:1355). Nations with similar economies tend to have similar features and incidences in these categories (Ackery *et al*. 2004:1355). Men are universally more likely to be injured than females and the incidence rates for males are consistently higher than for females for all age groups (Dryden *et al*. 2003:113). In Zimbabwe the male-female ratio was 8.1:1 (Levy *et al*. 1998:216) and in Bangladesh 7.5:1 (Hoque *et al*. 1999:859), whilst in high income countries the gender distribution was about 3.8:1 (Wyndaele & Wyndaele 2006:523). The reason for this might be that in low income countries the females are more often at home taking care of the family, whilst men are performing the risk-taking activities outside home and being more exposed to hazardous working environments and violent behaviour. The trend in high income countries indicates that women are slowly catching up (Wyndaele & Wyndaele 2006:527), whilst in low income settings men are still at a significant higher risk than women.

Globally, road traffic accidents involving motor vehicles, bicycles or pedestrians, accounted for about half of all SCIs, predominantly amongst young adults. Road traffic accidents accounted for 56% of all SCIs in Zimbabwe (Levy *et al*. 1998:216), 58% in Nigeria (Nwadinigwe *et al*. 2004), 42% in Brazil (Da Paz *et al*. 1992:636), 25% in South Africa (Hart & Williams 1994:709), and 18% in Bangladesh (Hoque *et al*. 1999:860). SCIs were most frequent in young adults between the ages of 16 to 30 years (54%) (DeVivo *et al*. 1999:1412), and 42% of persons with SCI were under 25 years of age at the time of the injury (Whiteneck *et al*. 1992:619). Alcohol and substance abuse is a potential risk factor for SCI.

Falls accounted for 63% of SCIs in Bangladesh and were split into two major causes: falling from a height, such as a tree (43%), and falling whilst carrying heavy loads on the head (20%) (Hoque *et al*. 1999:860). SCI caused by falling from a tree, which is experienced in many low income countries, can be explained by the agriculturally based economy, as adults and children climb trees to harvest fruits and to chop off tree branches for fire.

Figures from South Africa indicated a high incidence of SCI from violence (56%), particularly gunshot injuries (36%) and stab wounds (20%) (Hart & Williams 1994:709). This might be explained by socio-political changes and the general violence in the country (Hart & Williams 1994:709). In Brazil violence was the second leading cause accounting for 27% of SCIs (Da Paz *et al*. 1992:636). SCI from violence has been increasing in countries where violence is rife.

### Life expectancy after spinal cord injury

Before World War II people rarely survived for extended periods after a SCI (Krause, DeVivo & Jackson 2004:1764). Since then life expectancy has improved considerably and over the last three decades there has been a 40% decline in mortality during the critical first 2 years after injury in high income counties (Strauss *et al*. 2006:1079). In Zimbabwe, during the 1960s, approximately 90% of people with SCI in Zimbabwe died within one year of discharge from hospital (Levy *et al*. 1998:213). During the period 1988–1994, mortality as a result of SCI dropped and half of SCI patients who went through the National Rehabilitation Centre (NRC) in Ruwa, Zimbabwe, were surviving beyond one year (Levy *et al*. 1998:217). A high incidence of secondary complications resulted in a high mortality rate in hospitals.

Several studies on SCI indicate that higher neurological levels of injury, more severe degrees of completeness and higher ages at injury are increasing the risk of mortality (DeVivo *et al*. 1999:7416; Krause *et al*. 2004:1764; Krause *et al*. 2008:1482; Lidal *et al*. 2007:145; Whiteneck *et al*. 1992:617). In Zimbabwe two-thirds of those who died within one year after the injury were tetraplegics and one-third paraplegics (Levy *et al*. 1998:216). The chances of dying are highest in the first year after injury, particularly for severely injured persons. Ventilator dependency was the strongest predictor of mortality in the first year, and persons with tetraplegia had the highest rate of death from respiratory and digestive failure as well as from septicaemia. We were not able to identify any ventilator dependent informants for this study, and it is suggested that few ventilator dependent persons survived during the economic crises in Zimbabwe.

### Problems causing premature death

Until the 1990s renal failure and other urinary tract complications were reported to be the leading cause of death amongst persons with SCI in high income settings (DeVivo *et al*. 1999:1411; Krause *et al*. 2008:1483; Whiteneck *et al*. 1992:622). Significant advances in urologic management and prevention and treatment of pressure sores resulted in changes in the leading causes of death. A number of studies from high income countries over the past decades have revealed that pneumonia and other respiratory complications are now becoming the leading causes of death amongst people with SCI followed by septicaemia, urinary tract diseases, heart diseases and suicide (DeVivo *et al*. 1999:1411; Soden *et al*. 2000:607). Other studies also confirm this converging trend towards the situation in the general population of the leading causes of death being cardiovascular diseases and respiratory complications (Hagen *et al*. 2010:370; Lidal *et al*. 2007:145). In low income countries infections and septicaemia caused by urinary tract complications and pressure sores are still the leading cause of death for people with SCI (Gosselin & Coppotelli 2005:331; Hoque *et al*. 1999:859; Iwegbu 1983:83; Levy *et al*. 1998:213; Nwadinigwe *et al*. 2004:214). In Zimbabwe 7% of individuals with SCI died from septicaemia as a result of pressure sores whilst being hospitalised (Levy *et al*. 1998:214). High rates of pressure sores (33%) and pain (77%) after returning home were also documented (Levy *et al*. 1998:216-217). Pressure sores and resulting infections were experienced to be the major causes of death amongst persons with SCI in Zimbabwe.

In high income countries suicide rates are higher amongst persons with SCI than in the general population (DeVivo *et al*. 1999:1411; Soden *et al*. 2000:609), but similar findings could not be confirmed in this study or other studies from Zimbabwe (Levy *et al*. 1998:216). It might be that suicide is under-reported, and more careful exploration is needed.

#### Urinary tract infections

Paralysis from SCI can affect bladder and bowel functions depending on the extent of the injury. Bladder and bowel incontinence is a challenge for many people with SCI. Catheterisation increases the risk of infections if not carried out in a hygienic and safe environment. Many of the homes, in both urban and rural settings, do not have access to clean water or proper sanitation and this might easily cause infections. Lack of access to catheters and urinary bags increases the chances of having urinary tract infections. Stephen, a young man, stated: ‘I [*can*] hardly afford to buy urine bags and catheter[*s*] and they are hardly available now’ (I8, person with SCI, male). At R1.00 per day, disposable catheters are not costly: however for people living below the poverty line, it is money they do not have. Others used napkins and said: ‘If you have got a pressure sore and are using napkins that is … deadly’ (I1, person with SCI, female).

Education and training in how to do catheterisation is of major importance but unfortunately it is not available to all patients. Peter who had received training in South Africa explained:

‘I do disinfection of the catheter and urine bags every day, and I take out the catheter every day. This is very easy if you know how to do this. I also use some gel when inserting the catheter. I use sodium hypercloride for disinfection and I buy this at the pharmacy. You need to be educated about the catheterization. I was educated by the doctors in South Africa. But many doctors and nurses here are not trained.’ (I12, person with SCI, male)

Many of the informants and their families reported that they had not received adequate training for bladder and bowel management, which is probably a consequence of medical staff not being specially trained in caring for SCI patients.

#### Pressure sores

Pressure sores and ulcers, also called bed sores and decubitus ulcers, might easily occur when sitting or lying in the same position over time: for example lying in bed or sitting in an inappropriate wheelchair. It may, however, take just a few hours to get the first sign of pressure sores, and if not handled properly it might develop quickly. Many of the informants experienced pressure sores. These could take months to heal often causing infections and in worst cases death. Family members reported that the cause of death for three out of five individuals who passed away was from pressure sores, and one had pressure sores as a contributing factor. Rehabilitation professionals reported that infected pressure sores and urinary tract infections were causing major health problems amongst people with SCI, and are possibly still the leading cause of death. In Nigeria pressure sores were reported to be the most common complication (Nwadinigwe *et al*. 2004:161).

An older lady living in her rural home together with her family was paralysed in both legs after a fall. The family tried their best to take care of her, but because of the difficult economic situation and lack of access to health services and wheelchairs, it was difficult for her to recover after the injury. Her relatives explained:

‘Mama was given a wheelchair from hospital for a few days, but they needed it back when she could not pay for it. When she came home from hospital she developed pressure sores after one month, caused by staying in bed most of the time. She had sores all over, both at the side of the hips and inside the legs. At the end maggots were coming out of the wounds. Mama passed away in her home four months after she got the pressure sores.’ (I14, relative, female)

Sam became paraplegic at the age of 16 after having a spinal tumour removed. He used to live with his mother in a poor township. His mother stated:

‘After discharge, he was given a self-propelling wheelchair which had a plastic seat and this resulted in him having pressure sores on his back as soon as he settled back home. He was admitted to the hospital and the nurses would refuse to dress his wounds because they were so infected and he would pass urine as well as faeces without realizing it in bed. By then he had very bad pressure sores and a swollen left leg. He now needed everything done for him and after three days he died in the night and the family was told the following morning during visiting time.’ (I19, relative, female)

Emmanuel, who is living in an urban area with his wife and little son, stated:

‘I contracted pressure sores at hospital, and I spent six month in bed because of pressure sores. I got treatment for the pressure sores at the rehab centre. The medical doctors said I had a 50/50 chance to survive. But I made it and the pressure sores healed. I spent almost one year and three months at rehabilitation.’ (I7, person with SCI, male)

Pressure sores were reported to be a devastating complication, and a history of pressure sores was reported by many of the informants. Pressure sores might be contracted both at hospital and at home and are often caused by lack of turning and pressure relief routines and limited access to appropriate seating services and wheelchairs. Combined with limited access to health care services, poor hygienic conditions and a warm climate, pressure sores are a source of great suffering for people with SCI.

### Challenges of daily living

Most of the informants described the return home to the community as very difficult. One of the rehabilitation professionals summarised the situation:

‘When you are discharged from hospital and you are going home – going home to what? Often people with SCI do not have a wheelchair and the home conditions are not always conducive. The kinds of “bushtoilets” in rural area are not suitable for SCI, and sometimes you might not even have a bed. A person might be discharged from hospital to go home and die.’ (P17, rehabilitation professional, female)

#### Losing your job and limited access to health- and rehabilitation services

Out of 16 informants with SCI, 15 reported that they went home to their families after being discharged from hospital or rehabilitation. Limited income as a result of unemployment and, therefore, a lack of financial means to purchase devices such as wheelchairs, calipers, urinary bags and catheters, and limited access to transport and to health and rehabilitation services are some of the challenges restricting daily activities and social participation of people with SCI.

Unemployment is generally high in Zimbabwe, and informants reported that after their injuries they either lost their job or found it impossible to continue their work. Loss of employment meant loss of income which often had dramatic consequences for the households involved. Young people with SCI also reported that they had to leave university because of lack of access to school buildings and of accessible and affordable transport.

Hannah used to be the breadwinner of her family. They live in traditional houses about two to three kilometres from the main road in a rural setting. She cared for her mother and father, both blind, her son, her sister, her sister’s daughter and some young brothers. By the age of 25 she sustained a SCI from a road traffic accident and her legs were paralysed. Absence of income affected all the family members and her son had difficulties in attending school because of lack of money for school fees and uniforms. Hannah said:

Before the injury I was going to Zambia and getting things and selling them. I was also selling chickens and I used to be the breadwinner of the family. Now it is difficult to move around and carry things. Last year I nearly died from hunger. It is very difficult, because I cannot work like I did any longer. Even the cost of one dollar a term for school fees for my son, is challenging to pay. (I6, person with SCI, female)

The consequences of SCI were also dramatic for Emmanuel and his family. Emmanuel is living in an urban area with his wife and little son. He had a good job in a nationwide company and his own company car. After the accident he lost his job and the car. Emmanuel and his family had no regular income as a consequence of the injury. He was insured by his company, but the insurance was exhausted after rehabilitation. As a result of his SCI, Emmanuel and his family had no income, and although they lived in an urban area, close to health care facilities and rehabilitation services, they were not able to access the services because of loss of income and no accessible public transport. He explained:

‘I can’t afford to go the physio, because we do not have a car any longer and I can’t use public transport. My wife is doing the daily work in the house, taking care of our boy and assisting me. I need some drugs and vitamins, but I can’t afford to buy this. I can’t afford to do testing of urine in order to detect infections.’ (I7, person with SCI, male)

Persons living in rural areas had even greater challenges accessing rehabilitation services because of economic difficulties and transport problems. One of the rehabilitation professionals tried to paint the difficulties of accessing rehabilitation services:

‘Imagine yourself, if you are living in rural area and you are told to go to Ruwa (the NRC) next to Harare for rehabilitation. You don’t have money, you don’t have transport to Harare and sometimes you don’t even have a wheelchair for personal mobility, how could you possibly find your way to Ruwa?’ (I11, rehabilitation professional, female)

All informants except for one man, reported that they had serious economic challenges after their injury, affecting the standard of living for their families and also making it difficult to access health services. None of the informants received financial support from the government.

#### Lack of access to wheelchairs and services

It has been documented that only 18% – 36% of people with disabilities in southern Africa have access to assistive devices and services (Eide *et al*. 2011). Levy *et al*. (1998:215) summed it up when he said: ‘The wheelchair problem has haunted us for a very long time.’ Limited access to appropriate wheelchairs and services was also reported by many of the participants. All informants, except one elderly lady, had some kind of wheelchair or mobility device, but many of the wheelchairs were not properly adapted to either the individual user or the home environment.

Limited financial resources and lack of priorities have caused many low income countries to distribute wheelchairs through charity organisations and donations from abroad. Most of these wheelchairs have been designed to be used inside institutions or on even ground and are often not adapted to fit the environment or the individual user. The wheelchairs might only be available in one or a few sizes. Nena had sustained her SCI 15 years ago during a road accident and needed a wheelchair for both urban and rural areas. She highlighted the lack of appropriate wheelchairs adapted to the local environment: ‘In other countries there are different wheelchairs for SCI and lighter wheelchairs. Here in Zimbabwe we have to use any wheelchair’ (I1, person with SCI, female).

There is a huge need for a range of wheelchairs for various conditions. Nena explained that the rigid 3-wheeler wheelchair model was suitable for rural areas, but it was very difficult for her to use when travelling. The traditional foldable 4-wheeler was best suited for indoor use, for flat surfaces and for travelling, but it was too heavy for her to move in sandy areas, on dirt roads, up hills and around obstacles. Many of the informants in rural areas found it very difficult and sometimes impossible to push themselves from home to the main road or to attend social events or visit friends.

Fatuma, living in the rural area, explained that her first wheelchair from the Local Rehabilitation Workshop (LOREWO) in Bulawayo was strong and light and easy to use, but the donated orthopaedic style that she was currently using was very heavy and the small front castor wheels get stuck in the sand. She used to go to church once a week, but now with the donated wheelchair she found it almost impossible to push herself: ‘I can push myself, but now I have problems in the sand’ (I5, person with SCI, female). Nena added: ‘The donated wheelchairs look shiny, but they often break down after a short time and they are difficult to repair’ (I1, person with SCI, female).

It was observed that wheelchairs with even minor problems were not repaired because of a lack of spare parts and tools. Also, there was limited knowledge amongst users and professional rehabilitation staff on how to repair wheelchairs. During field work we experienced that wheelchairs had been donated without proper services and often not adapted to fit the individual user. Sam developed pressure sores that became infected and caused his death because of the wheelchair with plastic seating. An inappropriate wheelchair increases the risk of secondary complications like pressure sores, shoulder injuries or spinal deformities.

#### Pain and depression

Pain was a prominent factor in the lives of many SCI patients regardless of their level of injury and 77% of individuals with SCI complained about pain (Levy *et al*. 1998:217). High prevalence of chronic pain after SCI is also reported in high income countries, is likely to affect the quality of life, and may cause depression (Ravenscroft, Ahmed & Burnside 2000:611).

Findings from this study confirm that pain and depression are frequent. Pain is often permanent and one has to find a way of dealing with it and the dizziness. Matt, living with tetraplegia in an urban area elaborated as follows:

‘The pain never left me. This pain is different from anything else. [*I*]t is rootpain and pain in legs, numbness and burning as part of post traumatic. It is always worst in the evenings when it is cold. Ordinary pain killers like Voltaren do not work. Morphine like drugs does help, but they are not available and they are addictive. Anti-elliptic drugs are helpful. But from the sunset up to the night I have pain and burning. Pain is there.’ (I9, person with SCI, male)

Other major problems reported by individuals with SCI in Zimbabwe are boredom and lack of purpose (Levy *et al*. 1998:217). Many see themselves as totally useless and non-contributive, and unfortunately many of their families share this perception (Levy *et al*. 1998:214). A feeling of usefulness is important and one male informant with a severe SCI described his situation a year after the injury, where he also lost his wife, as follows:

‘I felt I was a hopeless mass of bone and flesh. I was supporting my children and talking to them, and then it was the first ray of light. I felt that a father in a wheelchair is better than no father. I would be alive for them (my children), this gave me the reason to live.’ (I9, person with SCI, male)

Samson, a paraplegic after a road traffic accident, was staying with his grandmother and grandfather. Because of his SCI he was not able to continue his education. He used to sit at home listening to music and commented: ‘What more can I do?’ (I8, person with SCI, male). Samson was bored at home and experienced that it was very difficult for him to contribute and become an active member of society. He emphasised the importance of meeting other people with disabilities and be inspired.

#### Stigma and attitudes

Many of the informants had experienced stigma or negative attitude from family members and society after their SCI. Peter is living in a rural area and was injured many years ago during a road traffic accident. He stated that:

‘Stigma is there, in the society, within family and in yourself. In order to handle stigma and negative attitudes, you have to accept your disability first and then you will be able to face society and fight stigma within family and society. Stigma is due to ignorance and lack of knowledge.’ (I 12, person with SCI, male).

A rehabilitation professional also referred to stigma: ‘Stigma is mostly within society. In earlier days a person with disabilities was just seen as a half person’ (I11, rehabilitation professional, female).

Nena confirms Peter’s negative experiences about attitudes towards people with SCI and also expresses her feelings of being useless after the injury. She described the traditional African culture:

‘Being an African woman is a lot of responsibilities. Many might think that you are not worth anything because you might not be able to carry out all the expected duties. If you fail to get counseling, you might just be staying at home. The attitude towards me changed after the injury. Even my husband did not like me to go out, and he used to get me things instead. Also the children said that they would not want me to go with them to school, because they would be embarrassed by the other children.’ (I1, person with SCI, female)

Luckily Nena received counselling and was trained. After some years she was able to accept her situation and could inform and train her family. She personally experienced how attitudes from people around her changed from negative to positive as her confidence and abilities grew. Many informants experienced a change in attitude in others when they were able to demonstrate their value, and when those around them learned more about SCI. The need for counselling and family support was emphasised by the informants.

#### Limited knowledge about SCI amongst medical staff

Lack of knowledge about SCI by health care staff at both urban and rural clinics and at central hospitals was experienced. Andrew who was injured during a sporting accident, complained on behalf of his friends with SCI:

‘Those who passed away, some of the cases I have to blame the nurses, because they are not doing their work, teaching the patients about pressure sores and handling of the bladder and bowel’ (I3, person with SCI, male)

Nena expressed her dissatisfaction with the care provided by hospital staff as follows:

‘I contracted pressure sores on my buttock and on my heels at hospital because of lack of turning. You might die in hospital because of pressure sores. Get out of hospital as early as possible. The pressure sores healed when I came home, because my sister and my husband dressed the sores three times a day. At hospital they do not do dressing three times a day.’ (I1, person with SCI, female)

Matt described his experiences like this:

‘MR scan showed that it was a lot of damage to the spine, C5/C7. I developed bronchia pneumonia and septicaemia. These are common complications. I had breathing problems, so breathing was a challenge. Bed sores happened in the sacral, and I had to spend one month at hospital in Harare. I lost tremendous weight. I am sure if it was a spinal unit here in Zimbabwe, I could have been much better. Then I was transferred to Pretoria with a proper spinal unit. There I could see a great difference in nursing care. They know how to look after bed sores. I got plastic surgery and started with physiotherapy.’ (I9, person with SCI, male)

Many of the informants are requesting adequate training of health care and rehabilitation professionals that would ensure better care for persons with SCI and furthermore improve their conditions.

## Discussion

Life expectancy for persons with SCI in high income countries has increased because of enhancements in medicine, prevention and treatment, including early acute management and long-term rehabilitation (DeVivo *et al*. 1999:1411; Krause *et al*. 2004:1764; Strauss *et al*. 2006:1079). Although the survival rate has improved also for individuals with SCI in low income countries, the mortality rate is still high and life expectancy is significantly lower than amongst the general population (Levy *et al*. 1998:213). A rehabilitation professional summed up her experiences: ‘Generally the poor ones die off due to lack of resources and health care. This seems to be the trend’ (I22, rehabilitation professional, female).

For high income countries significant improvements in the management of urologic issues and the prevention and treatment of pressure sores have shifted the leading causes of death to heart and cardiovascular diseases and respiratory complications. This shift in leading causes of death was not identified for low income countries, and in Zimbabwe septicaemia because of urinary tract infections and pressure sores and pneumonia had the greatest impact on life expectancy (Levy *et al*. 1998:214). The findings from this study also revealed that urinary tract infections and infectious pressure sores are frequently causing major health problems amongst people with SCI, possibly still being the leading cause of death. A high prevalence of pressure sores and pain after returning home was documented along with the need for wheelchairs (Levy *et al*. 1998:216-217). Sam died from infectious pressure sores caused by using an inappropriate wheelchair with plastic seating. Mama did not have a wheelchair and died from infectious pressure sores caused by lying in bed. Their problems might have been prevented if they had received appropriate wheelchairs and services. By constant turning and proper care, pressure sores could have been prevented or treated. Costly actions might not be necessary, just adequate training of carers, whether they are staff or family members, and ensuring the right knowledge and attitudes. Levy stated with reference to prevention and treatment of pressure sores that:

An enormous amount of money and time is spent trying to heal what is preventable. A ‘turning team’ whose job would be to go around any hospital turning all inert patients, would be of enormous benefit and save lives and money. (Levy *et al*. 1998:214)

If Sam and Mama had received proper care and rehabilitation services including appropriate wheelchairs, this could have facilitated survival instead of contributing to mortality. Access to financial support, health care- and rehabilitation services and appropriate wheelchairs might be alleviating factors improving the situation.

The situation for people with SCI in Zimbabwe was reported to have become worse because of the country’s economic decline. Collection of data for this study was conducted in 2009 during a time of economic recession in Zimbabwe and the near collapse of the health care system. This period of hyperinflation and economic breakdown presented most informants with SCI and their families with major challenges. They reported having no financial support, there were hardly any medical or technical services available, and many were not able to access rehabilitation services. It was illustrated that if you lived in a rural area, it might take days of travelling to get to the NRC: and how could you possibly get there without money and accessible transport? Generally persons with SCI were struggling to cope both at hospital and when returning to their communities. They reported that access to health care- and rehabilitation services was difficult, and mortality rate was high, possibly higher than in the 1980s.

Harry stated: ‘Poverty is the main problem’ (I4, person with SCI, male), and others echoed that poverty was the major challenge. Furthermore, Hannah explained how her family was struggling to survive because of hunger after her injury. Findings from this study confirm that poverty might accelerate the severity of disability after SCI. Stephen could not afford to purchase disposal catheters and urinary bags, and others did not have the resources to access rehabilitation services. As a consequence many contracted painful urinary tract infections and devastating pressure sores, which increased their disability and reduced their quality of life. Pressure sores were also contributing to premature death for four out of five individuals. Environmental barriers like limited access to mobility increased the daily challenges for people with SCI and hindered their access to services and participation in society.

The situation for persons with SCI in Zimbabwe was difficult and access to services, technical devices and social support was limited as a result of the economic recession and poverty in the country. The findings indicate that structural poverty in society might lead to a higher mortality rate because of limited access to adequate acute medical care and long-term rehabilitation services. Furthermore, poverty was experienced to have an even greater impact on persons with SCI and their families, as a result of the daily challenges presented by the low standard of living. Literature confirms that there is a link between poverty and disability, and being poor increases the likelihood of contracting secondary complications (Elwan 1999; Grut & Ingstad 2005; Yeo & Moore 2003).

Pain, boredom and depression were reported to have a major impact on the quality of life of people with SCI, both from the literature (Levy *et al*. 1998:217; Ravenscroft *et al*. 2000:611) and from this study. Matt stated, ‘[*n*]ot being able to walk is the least [*of my*] problems’ (I9, person with SCI, male), referring to the additional challenges like incontinence, infections, pressure sores, pain, the feeling of being useless, depression and stigma. Many informants reported developing mental stress and depression after the initial recovery phase and when returning home. Counselling by either professionals or peer groups and support from family were experienced to be factors contributing to increased self-esteem, mental health and participation. Many highlighted ‘‘[*m]*eeting others in the same situation, being inspired and have someone to talk to is important’ (I1, person with SCI, female), and the ‘[*c*]ounselling aspect is important, and the whole family needs to be involved’ (I2, person with SCI, male). Peer group training is relatively inexpensive and might be successfully implemented in low income areas. Exposing the society to people with SCI and demonstrate their ability was also experienced to reduce stigma and facilitate participation: ‘Make people feel useful’ (I9, person with SCI, male).

The findings indicate that structural poverty in a society worsens the situation for people with SCI by creating barriers, thereby increasing the risk of contracting harmful infections and pressure sores, and increasing mortality. The majority of the informants had a limited income because of losing their jobs and not receiving any financial support. They also experienced a lack of appropriate wheelchairs and affordable medical devices (catheter, urinary bags, etc.), limited access to public transport and education, limited social participation, mental stress and negative attitudes and stigma from family and society. These are all contributing factors reducing the quality of life and contributing to the mortality of people with SCI. Affordability and accessibility of services and devices, less prejudice and increased social participation might help facilitating survival of individuals with SCI.

A major challenge identified is a lack of income or financial resources supporting the individuals with SCI and their families. Levy (1998) described the challenges:

Unless we develop a very good financial and family support system and a strong personal drive we know we are virtually condemning that patient to death when we discharge them to out-patient care (p. 217).

His comments are still valid, reflecting the situation for many people with SCI in low income settings.

## Conclusions

The study indicates that life expectancy for individuals with SCI in less resourced settings is shorter than for the average population and also for persons with SCI in high income countries. Septicaemia as a result of urinary tract infections and pressure sores was still the leading cause of death and had the greatest impact on life expectancy and daily life amongst individuals with SCI. The findings confirm that individuals with SCI and their families in low income settings are facing major challenges in their daily life, because of limited financial, medical, social and technical support. Poverty has an even greater impact on people with SCI in low income countries, because of the daily challenges presented by the low standard of living. Poverty itself is contributing to individual suffering and a high mortality rate by limiting access to rehabilitation services including appropriate wheelchairs and medical treatment for complications like infections, pressure sores and respiratory problems, common to people with SCI.

Epidemiological studies of SCI in low income countries have not been identified (Wyndaele & Wyndaele 2006:252), and further explorations on mortality and how people with SCI and their families in low income settings are coping in their daily life are required to provide comprehensive evidences.

## Footnotes

1 English is not the first language of the informants and therefore some grammatical errors are present in their responses. To keep the authenticity of these responses, language errors were not corrected.

2 Incidence: average number per million inhabitants per year.

3 Prevalence: average number per million inhabitants.
